# The influence of modified Qing E Formula on the differential expression of serum exosomal miRNAs in postmenopausal osteoporosis patients

**DOI:** 10.3389/fphar.2024.1467298

**Published:** 2024-09-04

**Authors:** Junjie Lu, Hui Wu, Huan Jin, Ziyi He, Lin Shen, Chen Ma, Xiaojuan Xu, Zixian Wang, Bo Shuai

**Affiliations:** ^1^ Department of Integrated Traditional Chinese and Western Medicine, Union Hospital, Tongji Medical College, Huazhong University of Science and Technology, Wuhan, China; ^2^ Xiangyang Central Hospital, Affiliated Hospital of Hubei University of Arts and Science, Hubei Province, Xiangyang, China; ^3^ College of Sports Medicine, Wuhan Sports University, Wuhan, China

**Keywords:** postmenopausal osteoporosis, bone loss, MQEF; transcriptome sequencing, microRNA, bioinformatics analysis

## Abstract

**Objective:**

Although guidelines support the efficacy of Modified Qing’ E Formula (MQEF) in treating postmenopausal osteoporosis (PMOP), its underlying mechanisms remain incompletely understood. This retrospective investigation aims to elucidate MQEF’s impact on serum exosomal miRNA expression in postmenopausal osteoporosis patients and to explore potential therapeutic mechanisms.

**Methods:**

Following ethical approval and registration, postmenopausal osteoporosis patients aged 50–85 years, meeting the diagnostic criteria were randomly selected and received MQEF decoction supplementary therapy. Serum samples were collected pre- and post-treatment, followed by isolation and sequencing of exosomal miRNAs. Differential miRNAs in serum exosomes were identified, and bioinformatics analysis was conducted to discern the principal exosomal miRNAs involved in MQEF’s effects on PMOP and the associated signaling pathways.

**Results:**

Eighteen clinical blood samples were collected. A total of 282,185 target genes were detected across the three groups. 306 miRNAs exhibited altered expression in serum exosomes of PMOP patients, while MQEF intervention resulted in changes in 328 miRNAs. GO enrichment analysis revealed the immune and endocrine systems was pertained. KEGG enrichment analysis indicated associations between PMOP occurrence and MQEF treatment with cytokine interactions, oxidative phosphorylation, and the renin-angiotensin system. Intersectional analysis identified 17 miRNAs, including 2 consistent trends. miR-3188 as a potentially pivotal miRNA implicated in both PMOP occurrence and MQEF treatment.

**Conclusion:**

This study constitutes the first randomized, retrospective clinical exploration confirming that MQEF demonstrates regulatory influence over exosomal miRNA expression in PMOP patients’ serum, its impact likely involves modulation of the immune and endocrine systems, as well as the renin-angiotensin system.

## Introduction

Postmenopausal osteoporosis (PMOP), as an age-related condition, is increasingly prevalent in the global aging population, rendering PMOP a burgeoning concern worldwide (Walker and Shane, 2023; Compston et al., 2019; Black and Rosen, 2016). The neglect of PMOP may culminate in osteoporotic fractures, precipitating disability and mortality among the elderly, thus imposing significant economic and social burdens on healthcare systems (Ensrud and Crandall, 2024; Johnston and Dagar, 2020; Ayers et al., 2023). Current pharmacological interventions for PMOP typically categorize into anti-resorptive agents, bone formation promoters, and other mechanism-based drugs (Foessl et al., 2023; Reid and Billington, 2022). However, these medications demonstrate variable economic efficiency and potential side effects, presenting formidable challenges to PMOP management (Khosla and Hofbauer, 2017; Anam and Insogna, 2021). The imperative development of highly effective and minimally toxic anti-osteoporotic drugs is essential for their clinical applicability and widespread adoption.

The decline in ovarian function profoundly impacts skeletal disorders, with the mechanisms underpinning this inter-organ influence proving both intriguing and complex. Extensive research endeavors have focused on elucidating the substances and factors mediating inter-organ communication (Meczekalski et al., 2023; Costa et al., 2023). Extracellular vesicles, especially exosomes, serve as pivotal mediators of inter-organ communication due to their rich cargo of miRNAs, which exert profound biological effects by modulating gene expression and cellular functions in both health and disease (Gurung et al., 2021; He et al., 2018; Zhang et al., 2015). Exosomes facilitate cellular communication and material transfer between diverse tissues and organs, playing pivotal roles in the regulation of bone metabolism and homeostasis (Wang et al., 2022; Ju et al., 2023). Through the regulation of osteoblast and osteoclast differentiation, modulation of vascular endothelial growth factor expression, and regulation of the bone immune microenvironment, exosomes maintain a delicate balance between bone resorption and formation (Wang et al., 2023; Pishavar et al., 2021; Zhu et al., 2024). miRNAs, as the principal cargo of exosomes, represent a heavily scrutinized subgroup of small non-coding RNAs, whose biological properties have undergone extensive investigation in recent years (Shang et al., 2023; Diener et al., 2022). Given their evolutionarily conservative nature, miRNAs have garnered widespread attention for their roles as key regulatory factors in the pathogenesis and progression of skeletal disorders, including PMOP (Yang Y. et al., 2020; Leng et al., 2020).

Modified Qing’ E Formula (MQEF), first documented over 1,300 years ago and widely utilized in clinical practice, is recorded in ancient texts for its purported ability to bolster bones and enhance blood circulation. It has been integrated into the treatment guidelines for primary osteoporosis in China (Lu et al., 2022a). Despite MQEF’s millennium-long history of use, its mechanisms of action remain enigmatic. The theoretical underpinnings of its use stem from traditional Chinese medicine (TCM) concepts and clinical observations of efficacy. However, its clinical application heavily relies on the empirical knowledge of TCM practitioners, impeding its widespread acceptance. Modern pharmacological investigations into MQEF have unveiled its diverse pharmacological activities, including anti-inflammatory, anti-aging, and phytoestrogenic properties (Zhu S. et al., 2022; Zhong et al., 2016; Zhang et al., 2016). MQEF also enhances hepatic and renal function by modulating oxidative metabolism (Zhong et al., 2016). The active components and ratio of MQEF were summarized in detail by LC-MS/MS technique, which laid a foundation for the development of active components in the later stage. Our recent studies have demonstrated that MQEF augments vascular microcirculation in non-traumatic femoral head necrosis, mitigates inflammatory cytokine release, and promotes type H blood vessel formation through regulation of the renin-angiotensin-aldosterone system (RAAS), ultimately ameliorating bone microstructure and augmenting overall bone strength (Lu et al., 2022b). Nevertheless, the precise mechanisms underlying MQEF’s clinical efficacy in improving PMOP remain incompletely elucidated. Although its clinical efficacy is commendable, unraveling its putative molecular pharmacological mechanisms through scientific methodologies, and through the screening, isomerization optimization, and re screening of its effective active ingredients, then, invent clinically original drugs, represents a significant endeavor in advancing research into the modernization of TCM.

This study endeavors to scrutinize the specific molecular biological mechanisms through which MQEF clinically alleviates PMOP. Employing transcriptome sequencing technology and bioinformatics analysis, we discerned differential expression of circulating serum exosomal miRNAs pre- and post-MQEF treatment, delving into the biological processes in which they may be implicated. Our findings unveil substantial alterations in exosomal miRNAs within the circulating serum of PMOP patients, indicating their involvement in disease pathogenesis through modulation of the endocrine and immune systems, as well as interference with signal transduction and molecule interactions. MQEF intervenes in these targets by modulating cytokine interactions, oxidative phosphorylation, and the RAAS system, thereby participating in the regulation of bone metabolism and homeostasis in PMOP. This may represent one of the pivotal mechanisms underlying MQEF’s efficacy in alleviating PMOP.

## Materials and methods

### Ethical approval

This retrospective clinical investigation obtained approval from the Clinical Research Ethics Committee of Tongji Medical College, Huazhong University of Science and Technology, affiliated with Union Hospital. Prior to study commencement, all participants provided written informed consent, and it is registered on the national medical research registration and filing information platform (Filing number: MR-42-24–013815).

### Study design

**FIGURE 1:**
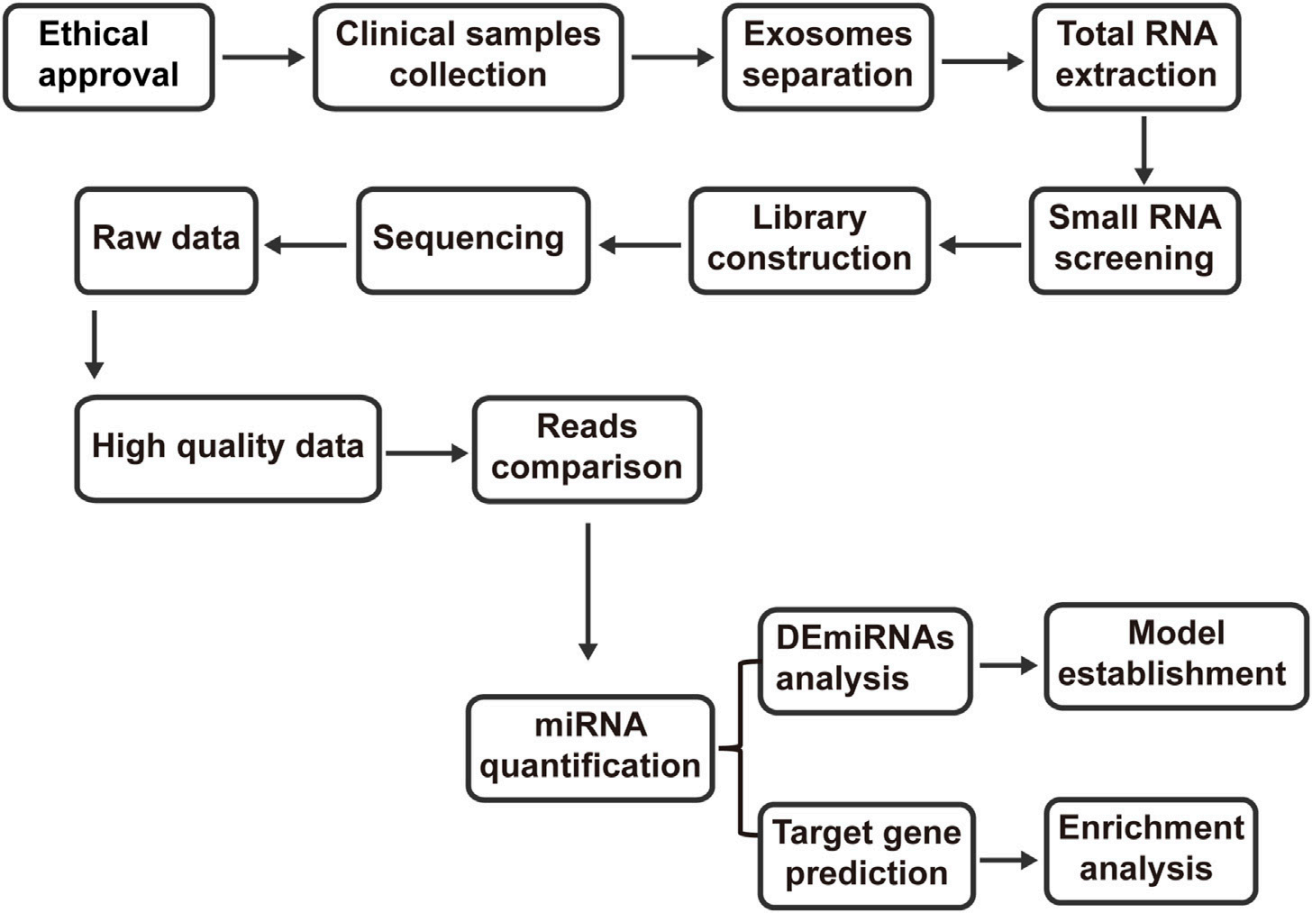
Study workflow of the present study.

A single-center, randomized controlled trial was conducted at the department of Integrated Traditional Chinese and Western Medicine, Union Hospital, Tongji Medical College, Huazhong University of Science and Technology. The trial comprised 12 PMOP patients and 6 healthy controls. Patient recruitment occurred among outpatients of the department of Integrated Traditional Chinese and Western Medicine, Union Hospital, Tongji Medical College, Huazhong University of Science and Technology. Any patient who met the diagnostic, inclusion, and exclusion criteria was eligible for enrollment. The clinical trial was considered to be over when the number of cases collected met the criteria and treatment and related data were collected. Subsequently, microRNA was extracted, exosomes were purified, and transcriptomics study was completed as the end of the whole study. Enrolled patients underwent screening for inclusion and confirmation of exclusion criteria before study enrollment. Using random number table method, the enrolled population was divided into different groups. Detailed inclusion and exclusion criteria for patients and healthy controls are provided in Table 1. The study workflow of the present study is shown in (Figure 1).

**TABLE 1:** Detailed inclusion and exclusion criteria for patients.

Inclusion criteria	Exclusion criteria
Voluntarily signing the Informed Consent Form, understanding the nature, purpose and experimental procedures of the research and willing to comply with the requirements of the research	Various metabolic bone diseases
Female, 50–85 years old (including 50 and 85 years old)	Paget’s disease
Absolute values of BMD in the lumbar spine (L1-L4) or total hip region of the patient are in accordance with: −4.0 < T-value ≤ −2.5	Cushing’s syndrome
Postmenopausal defined as menopause >2 years	Hyperprolactinemia
Chinese medicine symptoms consistent with deficiency of kidney yang	Thyroid Diseases
	Rheumatoid arthritis
	Malignant tumor
	Dysabsorption syndromes. e.g., Crohn’s disease and chronic pancreatitis
	Severe renal impairment; (Ccr <30 m/min)
	Hepatopathy
	Abuse of drugs or alcohol
	Various physical or mental illnesses
	Vitamin D deficiency
	Previously or currently using relevant drugs that affect bone metabolism
	Current hypocalcemia or hypercalcemia
	Abnormal liver transaminase
	The history of more than 2 vertebral fractures

### Intervention

Eligible PMOP patients underwent an additional venous blood collection of 5 mL on the first day of admission for routine examination after enrollment. Subsequently, patients received oral administration of MQEF decoction for 2 weeks, commencing between days 2 and 15 post-enrollment. A final venous blood collection of 5 mL was performed on the last day of treatment. The MQEF decoction comprised the following ingredients: *Eucommia ulmoides Oliv* (20 g), *Psoralea corylifolia Linn* (15 g), *Epimedium brevicornu Maxim* (10 g), *Rehmannia glutinosa Libosch* (10 g), *Achyranthes bidentata Blume* (10 g), *Salvia miltiorrhiza Bunge* (10 g). These herbal components were uniformly prepared into a 100 mL decoction by the department of herbal pharmacy, Union Hospital, Tongji Medical College, Huazhong University of Science and Technology, and stored at 4°C. MQEF was administered orally twice daily, with each dose being 100 mL, over a 2-week period.

### Exosome purification

Following venous blood collection and serum separation, the supernatant was obtained through centrifugation (3,000 g, 4°C, 15 min) and filtered through a 0.8 μm membrane before undergoing size-exclusion chromatography. The purification column was vertically fixed and equilibrated to room temperature. The waste collection tube was positioned beneath the exclusion column, and the top cover was removed, discarding the sealing liquid. An adapter was inserted, and 60 mL of sterile Dulbecco’s Phosphate-Buffered Saline (DPBS) was added. Afterward, the bottom cover was removed, and the column was washed with DPBS (flow rate approximately 0.5 mL/min). Following DPBS flow cessation, excess DPBS was aspirated using a pipette. Subsequently, 3 mL of the sample was added, and elution buffer (DPBS) was introduced. 1 mL was collected from each fraction, and the purified exosome solution was stored at −80°C for further use.

### The quality control of the extracted exosomes

The success of separating exosomes from clinical samples directly determines the reliability of subsequent experiments. According to the requirements of the International Society for Extracellular Vesicles for exosome identification: we performed protein marker identification (Supplementary Figure S1) on exosome samples. Western blot analysis was performed using the currently recognized and commonly used exosomes-specific markers TSG101 and Syntenin. Isolated exosomes were lysed by RIPA Lysis Buffer (Servicebio, Wuhan, China) with the addition of Phenylmethanesulfonyl fluoride (Servicebio, Wuhan, China). Protein loading buffer was added and protein denaturation was carried out at 95°C for 15 min. The protein samples were separated by 10% or 12% twelve alkyl sulfate polyacrylamide gel electrophoresis (SDS-PAGE) and transferred onto a PVDF membrane (Merckmilipore, Darmstadt, Germany). The PVDF membrane was blocked with 5% milk in TBST powder for 1 h and incubated with the primary antibody (TSG101/Syntenin, ABclonal Technology, Wuhan, China) at 4°C overnight. The membranes were washed with TBST three times, then incubated with HRP conjugated secondary antibody at room temperature for 1.5 h. After being washed with TBST for three times, membranes were incubated with ECL solution (Biosharp, Hefei, China), and the images were obtained with iBright CL750 imaging system (ChemiDoc MP, United States).

### Transcriptome sequencing

This stage was subcontracted to the GLW (Wuhan Genomics Institute), encompassing both experimental and analytical processes. The experimental procedure involved the following steps: total RNA underwent PAGE gel purification to isolate 18-30 nt RNA fragments, followed by ligation of 5′-adenylated, 3′-blocked single-stranded DNA adapters to the 3′end of the RNA fragments. Reverse transcription primers containing Unique Molecular Identifiers were introduced, hybridizing with the 3′RNA adapters and any excess free 3′adapters. Subsequently, 5′adapters were ligated to the 5′end of the resulting products, followed by reverse transcription extension using UMI-containing RT primers to synthesize single-stranded cDNA. High-fidelity polymerase was then employed to amplify cDNA with both 3′and 5′adapters simultaneously attached, thus increasing yield. PCR products within the 110-130 bp range underwent PAGE gel electrophoresis, followed by library quantification, pooling, and circularization. The quality of constructed libraries was assessed, and those meeting quality control standards underwent sequencing. Raw sequencing data underwent quality control and filtering to obtain high-quality data. Reads obtained from sequencing were aligned to the reference genome, and miRNAs and other non-coding RNAs were predicted based on the genomic positions of the sequencing data. Quantification of miRNAs and differential expression analysis were conducted, and all analysis results were uploaded to the Dr. Tom system for cloud-based analysis (https://biosys.bgi.com/. Project number: F23A040000899_HOMmgcoS).

### Basic information

A total of 18 samples were sequenced using the DNBSEQ platform, with an average output of 27.84 million data per sample, detecting a total of 1,130 small RNAs. The average alignment rate of samples to the genome was 89.46%, with the reference genome version being GCF_000001405.39_GRCh38. p13. The experiment comprised three groups, each containing 6 biological replicates. Pairwise comparisons were conducted between the healthy control, PMOP, and MQEF groups. Pearson correlation coefficients of expression levels for all genes between each pair of samples were calculated and visualized as a heatmap to reflect gene expression correlations. Boxplots were generated to observe data dispersion in expression level distribution. Density plots of miRNA expression were created to illustrate trends of gene abundance with changes in expression levels across samples. A stacked bar chart representing miRNA count statistics across different TPM intervals was generated for visual representation. To ensure result reliability, reads were filtered to eliminate low-quality reads, adapter contamination, and reads with high unknown base (N) content (Supplementary Figure S2 and Supplementary Tables S1, S2).

### Bioinformatics analysis

The Genomic Institute’s Dr. Tom system was utilized for bioinformatics analysis. The threshold for differential expression of miRNAs was set at log2FC > 2, *p* < 0.05. Differential expression miRNAs were obtained from various miRNA target gene databases, such as miRTarBase and TargetScan, to construct mRNA regulatory networks. Cytoscape was employed for analysis and visualization purposes. The Least Absolute Shrinkage and Selection Operator (LASSO) algorithm was applied to select statistically significant model features.

## Results

### Differential expression analysis of miRNAs in circulating blood exosomes among the three groups

**FIGURE 2:**
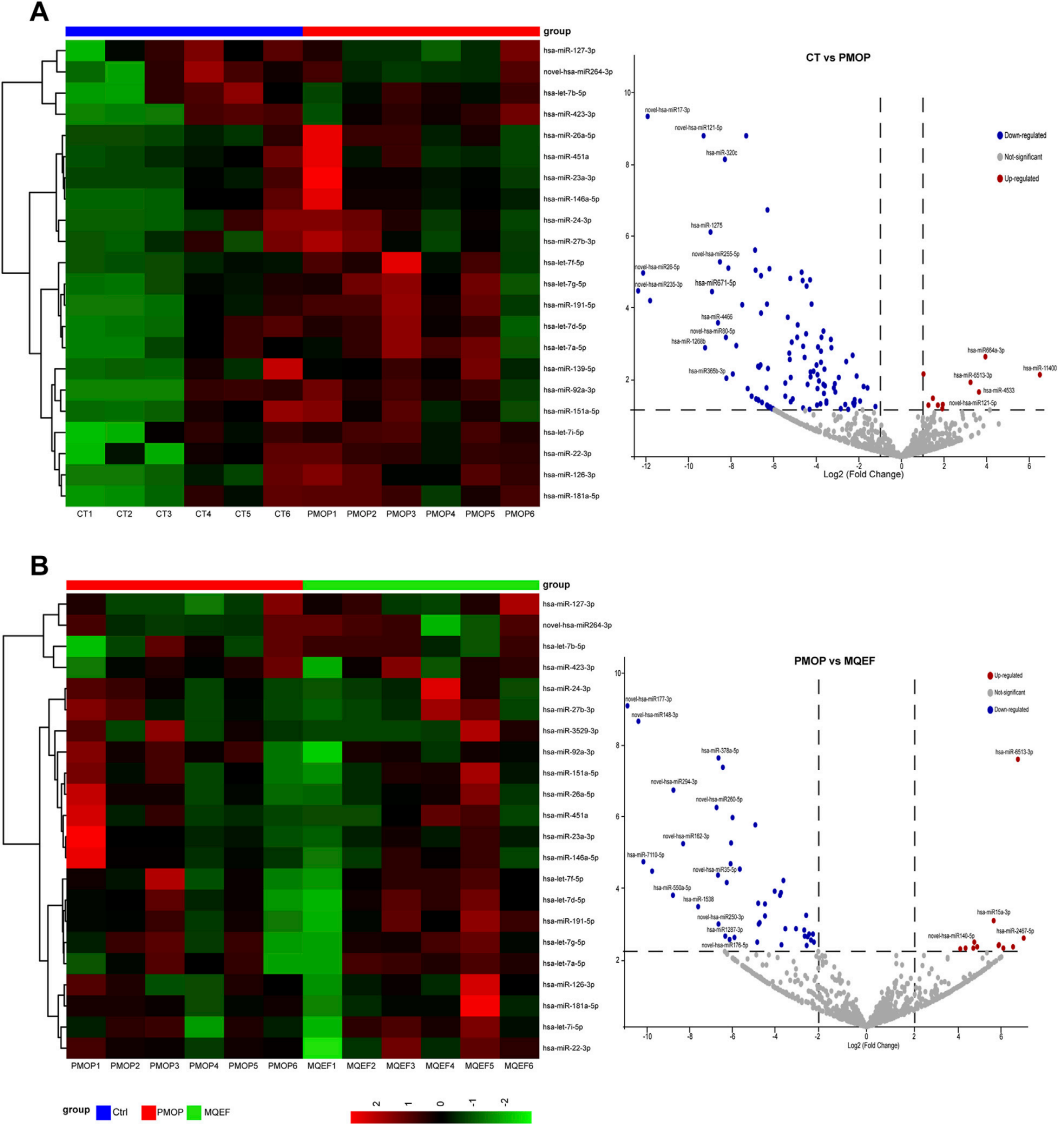
Differential miRNA display. **(A)** Differential miRNA heat map and volcano plot between the control group and PMOP group. **(B)** Differential miRNA heat map and volcano plot between the PMOP group and MQEF group. Differential expressed miRNAs were sorted by LogFc and *P* value.

Compared to healthy controls, individuals with PMOP exhibited 271 upregulated miRNAs and 192 downregulated miRNAs in serum exosomes. Following intervention with MQEF, 179 miRNAs were upregulated and 231 were downregulated in PMOP patients. Visualization of these differential miRNAs was achieved through volcano plots and heatmaps (Figure 2).

### Enrichment analysis of predicted target genes of differentially expressed miRNAs

**FIGURE 3:**
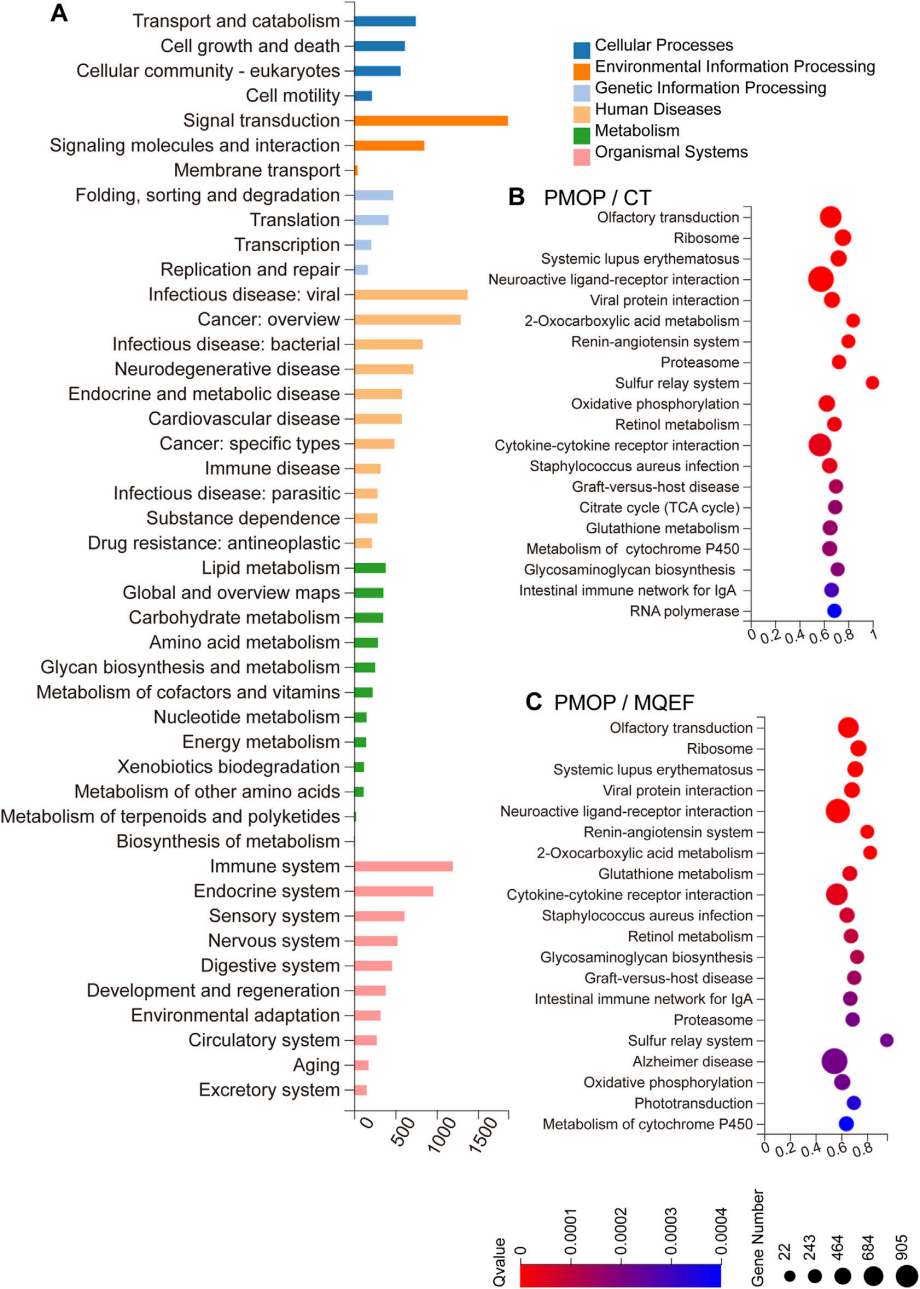
GO and KEGG Enrichment. **(A)** Biological processes involved in the differentially expressed miRNA-regulated predicted target genes. **(B,C)** KEGG pathway sorted by enrichment ratio between three different group.

Gene Ontology (GO) enrichment analysis revealed that the predicted target genes regulated by differentially expressed miRNAs were primarily involved in modulating the immune and endocrine systems, participating in signal transduction, and molecular interactions by influencing endocrine and metabolic processes (Figure 3A). Kyoto Encyclopedia of Genes and Genomes (KEGG) enrichment analysis indicated that both PMOP progression and MQEF intervention involved cytokine interactions, oxidative phosphorylation, and activation of the RAAS (Figures 3B, C). These findings align with our previous research conclusions. Our prior investigation illustrated that MQEF inhibits RAAS system activation, fosters type H vessel formation, thereby mitigating glucocorticoid-induced osteoporosis in murine models (Lu et al., 2022b). Additionally, in a mouse model of hormone-induced femoral head necrosis, we isolated exosomal miRNAs, conducted sequencing, and observed consistent results with our clinical study, thereby reinforcing the reliability of our findings (Zhu W. et al., 2022).

### Trend analysis and model validation

**FIGURE 4:**
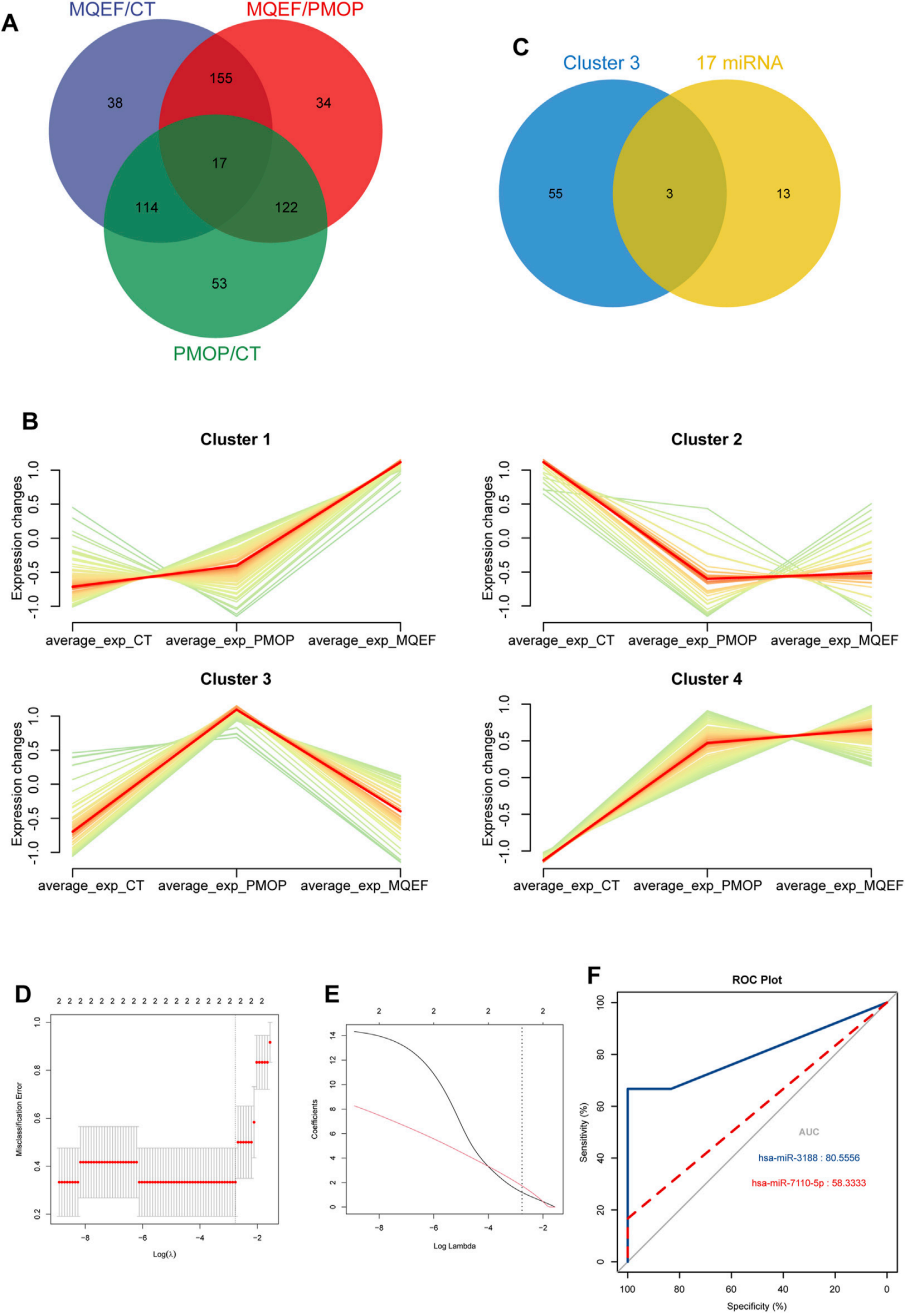
The hub miRNAs analysis and the model establishment. **(A)** The overlapped miRNAs from three different group. **(B)** The different trends of miRNAs expression in different clusters in three groups. **(C)** The overlapped miRNAs from the 17 differential expressed miRNAs and cluster 3. **(D)** The optimal parameter (λ) selection in the least absolute shrinkage and selection operator model used five-fold cross-validation via minimum criteria. The partial likelihood deviation (binomial deviation) curve was plotted against log(λ). A dashed vertical line was drawn at the optimal value using the smallest criterion and 1 SE of the smallest criterion (1-SE criterion); **(E)** A coefficient distribution map was generated for log(λ), where the optimal λ was the two features with nonzero coefficients. **(F)** Model validation receiver operating characteristic curve.

**FIGURE 5:**
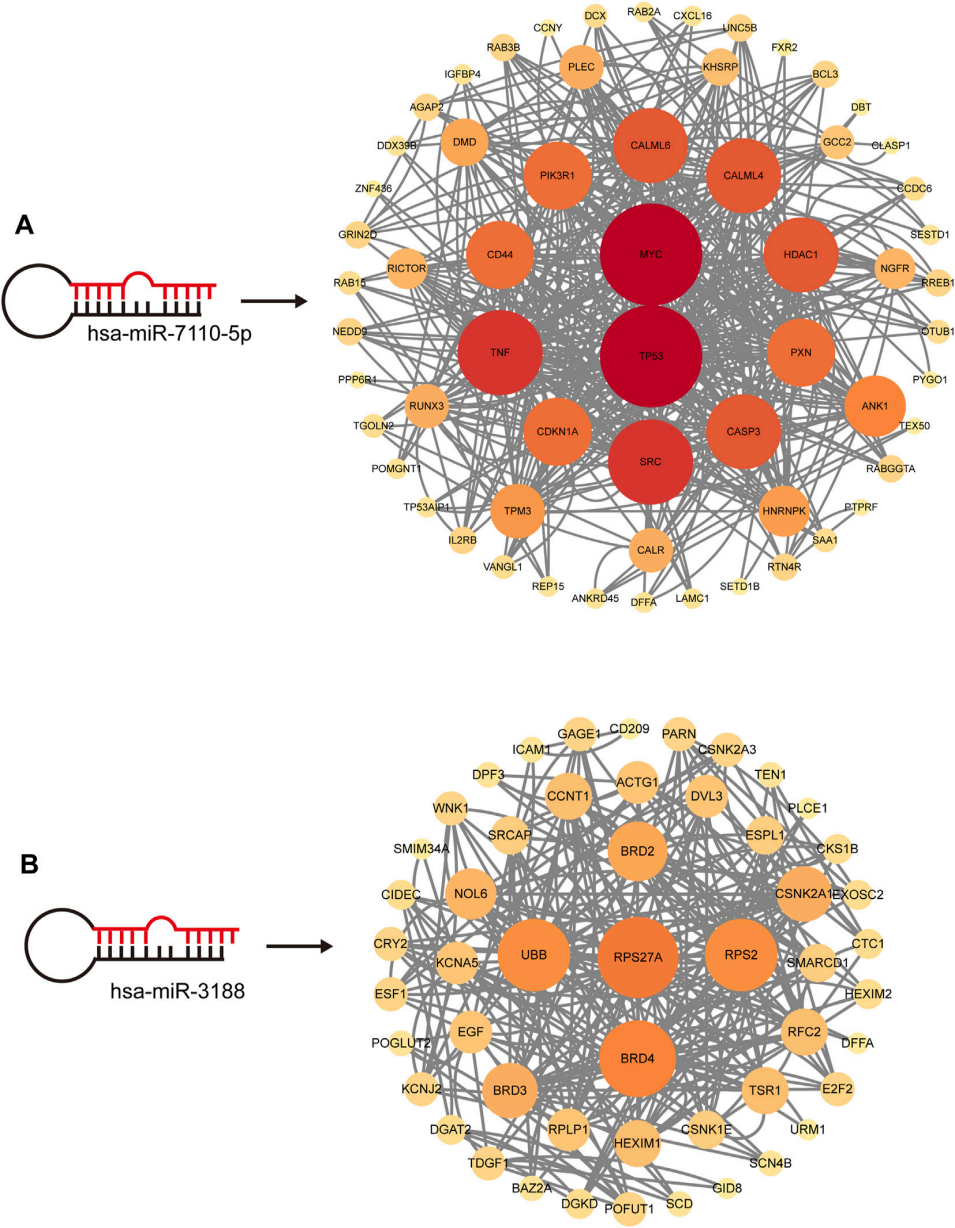
The Hub miRNAs targeted mRNA network. The size and color of the circle are enhanced according to its position in the interaction network. **(A)** hsa-miR-7110-5p targeted mRNA network. **(B)** hsa-miR-3188 targeted mRNA network.

Through joint comparative analysis of differential miRNAs among healthy controls, PMOP, and MQEF intervention groups, we identified 17 differential miRNAs (Figure 4A). Clustering trend analysis was performed on these miRNAs to identify those exhibiting a V-shaped trend, with cluster 3 containing miRNAs manifesting the desired trend (Figure 4B). Intersection of these miRNAs with the 17 miRNAs obtained from group comparisons yielded 2 differential miRNAs (Figure 4C). Subsequently, two predictive models were established for these miRNAs to identify the miRNA with optimal efficacy (Figures 4D–F). Analysis revealed that miR-3188 exhibited the desired trend and was highly associated with the onset of PMOP and the therapeutic effect of MQEF. By predicting target genes of these miRNAs and constructing a gene regulatory network, we confirmed that miR-7110–5p and miR-3188 exert regulatory effects on multiple mRNAs (Figure 5).

## Discussion

PMOP poses a significant public health challenge, especially with the escalating global aging population. A meta-analysis involving 103, 334, 579 individuals revealed a worldwide osteoporosis incidence of 18.3 (95% CI 16.2–20.7), with females exhibiting an incidence of 23.1 (95% CI: 19.8–26.9) (Salari et al., 2021). In China, 20.6% of women aged 40 and above suffer from osteoporosis, yet only 1.4% receive or undergo anti-osteoporosis treatment (Wang et al., 2021). Females post-menopause are more prone to osteoporosis compared to males. Aging triggers persistent low-grade immune system activation, fostering a pro-inflammatory milieu. Prolonged release of inflammatory mediators activates osteoclasts while suppressing osteoblasts, leading to bone loss (Fischer and Haffner-Luntzer, 2022; Wu et al., 2021). Age-related muscle wasting and reduced physical activity further impede the mechanical stimulation vital for bone remodeling (Adejuyigbe et al., 2023; Song et al., 2022; Yang T. L. et al., 2020). Declining estrogen levels weaken osteoclast inhibition, exacerbating bone resorption. Additionally, decreased antioxidant capacity compromises the body’s ability to counter oxidative stress, resulting in apoptosis of mesenchymal stem cells and osteoblasts (Raisz, 2001; Lu and Tian, 2023; Väänänen and Härkönen, 1996). PMOP is thus a multifactorial outcome. As a silent threat, early-stage PMOP often lacks discernible symptoms, complicating detection and treatment. Limited diagnosis and treatment hinder early intervention, with PMOP typically garnering attention only in advanced stages, culminating in compromised bone strength or fractures, commonly in the vertebrae and hip joints, leading to chronic pain or disability. Osteoporotic fractures constitute a primary cause of disability and mortality among the elderly, drastically reducing their quality of life and escalating caregiving costs. Within a year of hip fracture, 20% of patients succumb to complications, underscoring the substantial human and economic burden imposed by osteoporosis and its complications worldwide (Johnston and Dagar, 2020; Khosla and Hofbauer, 2017; Słupski et al., 2021; Lorentzon, 2019). Hence, proactive prevention and treatment pre-fracture are imperative, with active intervention mitigating fracture risk and effective post-fracture treatment reducing recurrence.

In recent decades, substantial strides have been made in understanding osteoporosis pathogenesis and developing novel treatments. Presently, anti-osteoporosis drugs predominantly comprise three categories: bone formation promoters, bone resorption inhibitors, and agents with alternative mechanisms. Common bone resorption inhibitors encompass bisphosphonates, hormone replacement therapy, and calcitonin. Bone formation promoters include parathyroid hormone analogs, fluoride, growth hormone, and statins. Other mechanism drugs entail strontium salts, vitamin D, vitamin K, and TCM (Black and Rosen, 2016; Foessl et al., 2023; Lu et al., 2022a; Li et al., 2021; Langdahl, 2021). With widespread utilization of large-scale sequencing technologies, an increasing array of PMOP-related targets has been identified for neutralizing antibody development. These antibodies exhibit marked differences from conventional drugs, binding selectively to receptors for precise regulation. Denosumab selectively inhibits RANKL, impeding osteoclast differentiation and formation (Zaheer et al., 2015). Romosozumab targets SOST, preserving bone homeostasis via wnt signaling pathway maintenance (Wu et al., 2023). Odanacatib inhibits Cat K, curbing bone resorption and collagen degradation (Chen et al., 2023). The first two neutralizing antibodies have FDA approval for PMOP clinical use, with Odanacatib in phase III trials. Antibodies targeting other antigens are under development or in preclinical trials. While appreciating these advancements, reflection reveals that most clinically utilized PMOP therapies predominantly target bone resorption or osteoclast function, with few promoting bone formation. Additionally, existing therapies are constrained by limitations, such as transient allergic reactions and renal impairment from bisphosphonates, and osteonecrosis of the jaw and rebound vertebral fracture risk post-Denosumab discontinuation. In sum, no single drug is flawless, and a diverse armamentarium offers clinicians broader therapeutic options.

Exosomes, as essential mediators of intercellular communication and material transfer, exhibit diverse functionalities, encompassing tissue regeneration and immune regulation. Their involvement in cell proliferation and differentiation has drawn considerable interest within the skeletal system (Ju et al., 2023; Yin et al., 2021). Studies suggest that exosomes wield influence over the bone microenvironment *in vivo*, exerting regulatory effects on various cell types, including osteoblasts, osteoclasts, osteocytes, chondrocytes, vascular endothelial cells, and immune cells. Cells within the bone microenvironment release exosomes containing miRNAs, which modulate both bone formation and resorption (Liu et al., 2017; Li et al., 2019). Exosomal miRNAs can regulate osteogenic-related mRNA expression, either promoting or inhibiting the proliferation and differentiation of osteoblasts, and similarly influence osteoclast-related mRNA expression to promote or inhibit osteoclast proliferation and differentiation. The exploitation of this dual regulatory capacity holds promise for mitigating pathological imbalances in bone metabolism, thereby restoring equilibrium between bone formation and resorption. Additionally, exosomes offer advantages as cell-free therapies, characterized by low immunogenicity and an absence of safety and ethical concerns. Their minute size (40–100 nm) facilitates penetration through various barriers, enabling entry into capillaries and overcoming multiple obstacles, positioning exosomes as an auspicious therapeutic avenue (Lai et al., 2022; Yang et al., 2019). However, imperfections such as the need for enhancement in large-scale isolation and purification techniques, short half-life, rapid metabolism, and potential off-target risks present challenges to clinical translation.

The biological effects of exosomes hinge on the miRNAs they carry, which exert potent regulatory effects by targeting hundreds of mRNAs individually. The classical function of miRNAs involves binding to the 3′untranslated region of sequence-specific mRNAs, resulting in mRNA translation inhibition or degradation (Ferragut Cardoso et al., 2021; Rupaimoole and Slack, 2017). Given their robust regulatory role, miRNAs are indispensable for normal cellular activity, and their dysregulation precipitates disease, including within the skeletal system. MiRNAs participate in numerous biological processes in bone metabolism, including osteoblast differentiation, angiogenesis, and immune microenvironment regulation, underscoring their pivotal role in modulating bone metabolism bioactivity (Taipaleenmäki, 2018; Gao et al., 2020). With the ongoing advancement of genomic technologies, understanding of diseases deepens progressively. Therapies targeting specific RNAs for therapeutic purposes offer the potential for precise treatment without the need for complex synthesis processes. Nonetheless, RNA-based drugs encounter challenges such as poor stability, large size, and charged properties, precluding intercellular delivery. Consequently, miRNAs that bind specifically to certain mRNA sequences garner significant attention. Therapeutic miRNA therapy is nascent in development, exemplified by MRX34, a lipid formulation of miR-34a, recognized as the first miRNA mimic used to treat multiple cancers. Currently undergoing phase I clinical trials, MRX34 holds promise (Zhang et al., 2019; Ling et al., 2017). However, miRNA-based therapies are still in their infancy, and overcoming challenges such as enhancing targeting specificity and stability, reducing toxicity and immunogenicity, optimizing bioavailability, and achieving organ-specific delivery remain formidable obstacles.

Over the course of more than 3,000 years, TCM has amassed a wealth of clinical experience and efficacious prescriptions for managing osteoporosis. Despite the absence of a specific term for osteoporosis in TCM theory, numerous treatments within the TCM paradigm have demonstrated effectiveness in ameliorating its symptoms. Among these treatments, MQEF stands out due to its extensive historical use and well-defined clinical efficacy. Employed in conjunction with both Chinese and Western medical approaches, MQEF adeptly mitigates clinical manifestations such as lower back pain and muscle spasms associated with osteoporosis. Owing to its sustained clinical efficacy, MQEF has been incorporated into guidelines for the diagnosis and treatment of primary osteoporosis. According to TCM principles, osteoporosis patients commonly present with two primary pathological features: “deficiency” and “stasis.” Therapeutic interventions predominantly center on tonifying kidney and nourishing essence and activate blood circulation and remove blood stasis. Comprising six medicinal ingredients, MQEF not only relieves lumbar discomfort and muscle spasms but also enhances lower limb strength and systemic microcirculation. Only a small number of patients had occasional gastrointestinal adverse reactions, such as abdominal distension or dyspepsia after taking MQEF, which could recover spontaneously after drug withdrawal without special treatment. This adverse reaction may be related to gastrointestinal dysfunction caused by drug stimulation. Short-term MQEF administration did not produce liver and kidney function damage, and the potential adverse effects of long-term MQEF treatment were not investigated because the clinical use of MQEF usually does not exceed 3 months. Clinical investigations have elucidated MQEF’s diverse pharmacological activities, including anti-inflammatory, anti-aging, and estrogenic effects. Furthermore, studies have shown MQEF’s capacity to ameliorate local hypercoagulability and hemodynamic imbalance in patients with non-traumatic osteonecrosis of the femoral head, reducing inflammation and elevating serum levels of bone sclerosis proteins (Li et al., 2017). Following MQEF intervention, improvements in bone mineral density at the femoral head and lumbar vertebrae have been observed in patients with primary osteoporosis (Yang et al., 2015). However, the clinical modulation of miRNA expression by MQEF in patients with primary osteoporosis remains unexplored.

This study elucidates one mechanism by which MQEF exerts its therapeutic effects on primary PMOP through the modulation of extracellular vesicle miRNAs, resulting in the upregulation and downregulation of specific miRNAs implicated in PMOP attenuation. Enrichment analysis further underscores the involvement of immune and endocrine system regulation in this alleviation mechanism, achieved through the modulation of signal transduction and interactions among signaling molecules. Specifically, MQEF may alleviate PMOP by engaging in cytokine interactions, oxidative phosphorylation, and regulation of the RAAS. These findings align closely with current animal experimental results, which indicate that MQEF intervention mitigates RAAS system activation and facilitates the generation of H-type vessels, thereby enhancing the bone microenvironment in hormone-induced osteoporosis animal models. In models of hormone-induced femoral head necrosis, MQEF intervention targets extracellular vesicle miRNAs, impacting immune and endocrine system function and signaling pathways associated with bone metabolism. Additionally, two key differentially expressed miRNAs have been identified and validated, paving the way for further exploration of MQEF’s molecular mechanisms in PMOP alleviation.

## Conclusion

To summarize, this study elucidates, for the first time, one of the mechanisms underlying the therapeutic efficacy of MQEF in managing PMOP at the clinical level, specifically through the targeting of exosomal miRNAs. Through the modulation of specific miRNA expression, MQEF exerts influence on immune and endocrine system functions, engages in signal transduction, cytokine interactions, oxidative phosphorylation, and the RAAS system, thereby mitigating the pathological progression of PMOP. Through a rigorous exploration of MQEF’s molecular biology and contemporary pharmacological mechanisms, this study furnishes compelling evidence to bolster the clinical utilization of MQEF.

## Data Availability

The original contributions presented in the study are included in the article/Supplementary Material, further inquiries can be directed to the corresponding author.

## Supplementary material

SUPPLEMENTARY FIGURE S1 The quality control of the extracted exosomes. The expression of several exosome-specific markers TSG101 and Syntenin were detected using Western blotting.

SUPPLEMENTARY FIGURE S2 The Data filtering and quality control. **(A)** Pearson correlation coefficients were calculated for all miRNAs expression levels between every two samples, and these coefficients were reflected by heatmaps. **(B)** Boxplots show the distribution of miRNAs expression levels in each sample. **(C)** The density map can show the trend of miRNAs abundance in the sample as the expression level changes. **(D)** The distribution of small RNA lengths.
