# Supplementary material for: The influence of modified Qing E Formula on the differential expression of serum exosomal miRNAs in postmenopausal osteoporosis patients
Source: Front Pharmacol. 2024 Sep 4;15:1467298. doi: 10.3389/fphar.2024.1467298 (PMC11408320; doi:10.3389/fphar.2024.1467298)
Supplement: Supplementary file 1 [file Table1.docx]

**Supplementary table 1. Quality statistics of filtered Reads (small RNA).**

| Sample | Sequence Type | Raw Tag Count | Low Quality Tag Count | Invalid Adapter Tag Count | PolyA Tag Count | Short Valid Length Tag | Clean Tag Count | Q20 of Clean Tag | Percentage of Clean Tag (%) |
| --- | --- | --- | --- | --- | --- | --- | --- | --- | --- |
| CT1 | SE50 | 2.80E+07 | 88952 | 3006997 | 4321 | 364662 | 2.45E+07 | 97.3 | 87.62 |
| CT2 | SE50 | 3.08E+07 | 111580 | 2714241 | 9653 | 3877052 | 2.41E+07 | 97.3 | 78.18 |
| CT3 | SE50 | 2.79E+07 | 100558 | 1552458 | 9670 | 1902404 | 2.44E+07 | 97.2 | 87.23 |
| CT4 | SE50 | 2.84E+07 | 104180 | 770895 | 447 | 637622 | 2.69E+07 | 97.1 | 94.67 |
| CT5 | SE50 | 2.99E+07 | 87531 | 908922 | 909 | 625535 | 2.83E+07 | 97.4 | 94.58 |
| CT6 | SE50 | 2.80E+07 | 95098 | 662944 | 311 | 195794 | 2.70E+07 | 97.2 | 96.59 |
| PMOP1 | SE50 | 2.92E+07 | 97391 | 740350 | 482 | 436596 | 2.79E+07 | 97.3 | 95.63 |
| PMOP2 | SE50 | 2.99E+07 | 110420 | 825053 | 550 | 226505 | 2.87E+07 | 97.3 | 96.11 |
| PMOP3 | SE50 | 3.01E+07 | 124725 | 859142 | 1017 | 129899 | 2.90E+07 | 97.1 | 96.3 |
| PMOP4 | SE50 | 2.97E+07 | 131077 | 1845362 | 804 | 125181 | 2.76E+07 | 97.1 | 92.92 |
| PMOP5 | SE50 | 2.87E+07 | 128432 | 572539 | 717 | 35828 | 2.80E+07 | 98.1 | 97.43 |
| PMOP6 | SE50 | 2.82E+07 | 129039 | 623244 | 522 | 91379 | 2.74E+07 | 98 | 97.01 |
| MQEF1 | SE50 | 2.89E+07 | 103160 | 797209 | 1604 | 189896 | 2.78E+07 | 97.3 | 96.22 |
| MQEF2 | SE50 | 2.99E+07 | 121853 | 531529 | 1015 | 33500 | 2.92E+07 | 97.2 | 97.7 |
| MQEF3 | SE50 | 3.02E+07 | 109173 | 625748 | 592 | 67718 | 2.94E+07 | 97.4 | 97.34 |
| MQEF4 | SE50 | 2.83E+07 | 125020 | 707401 | 1412 | 81788 | 2.74E+07 | 96.9 | 96.77 |
| MQEF5 | SE50 | 3.00E+07 | 111396 | 592241 | 592 | 171826 | 2.91E+07 | 97.4 | 97.08 |
| MQEF6 | SE50 | 2.95E+07 | 108812 | 580418 | 758 | 109861 | 2.87E+07 | 97.3 | 97.28 |

**Supplementary table 2. Reference genome alignment.**

| Sample | Total Clean Reads (M) | Total Mapping(%) |
| --- | --- | --- |
| CT1 | 23.38 | 81.14 |
| CT2 | 22.94 | 67.31 |
| CT3 | 23.23 | 83.47 |
| CT4 | 25.61 | 91.13 |
| CT5 | 27.01 | 87.91 |
| CT6 | 25.79 | 92.58 |
| MQEF1 | 26.51 | 86.24 |
| MQEF2 | 27.81 | 91.85 |
| MQEF3 | 27.99 | 93.1 |
| MQEF4 | 26.16 | 92.24 |
| MQEF5 | 27.76 | 93.42 |
| MQEF6 | 27.33 | 92.13 |
| PMOP1 | 26.59 | 93.26 |
| PMOP2 | 27.41 | 93.21 |
| PMOP3 | 27.67 | 92.25 |
| PMOP4 | 26.31 | 91.31 |
| PMOP5 | 26.66 | 94.27 |
| PMOP6 | 26.13 | 93.54 |
|  |  |  |
