# Supplementary figures and images for: The influence of modified Qing E Formula on the differential expression of serum exosomal miRNAs in postmenopausal osteoporosis patients
Source: Front Pharmacol. 2024 Sep 4;15:1467298. doi: 10.3389/fphar.2024.1467298 (PMC11408320; doi:10.3389/fphar.2024.1467298)

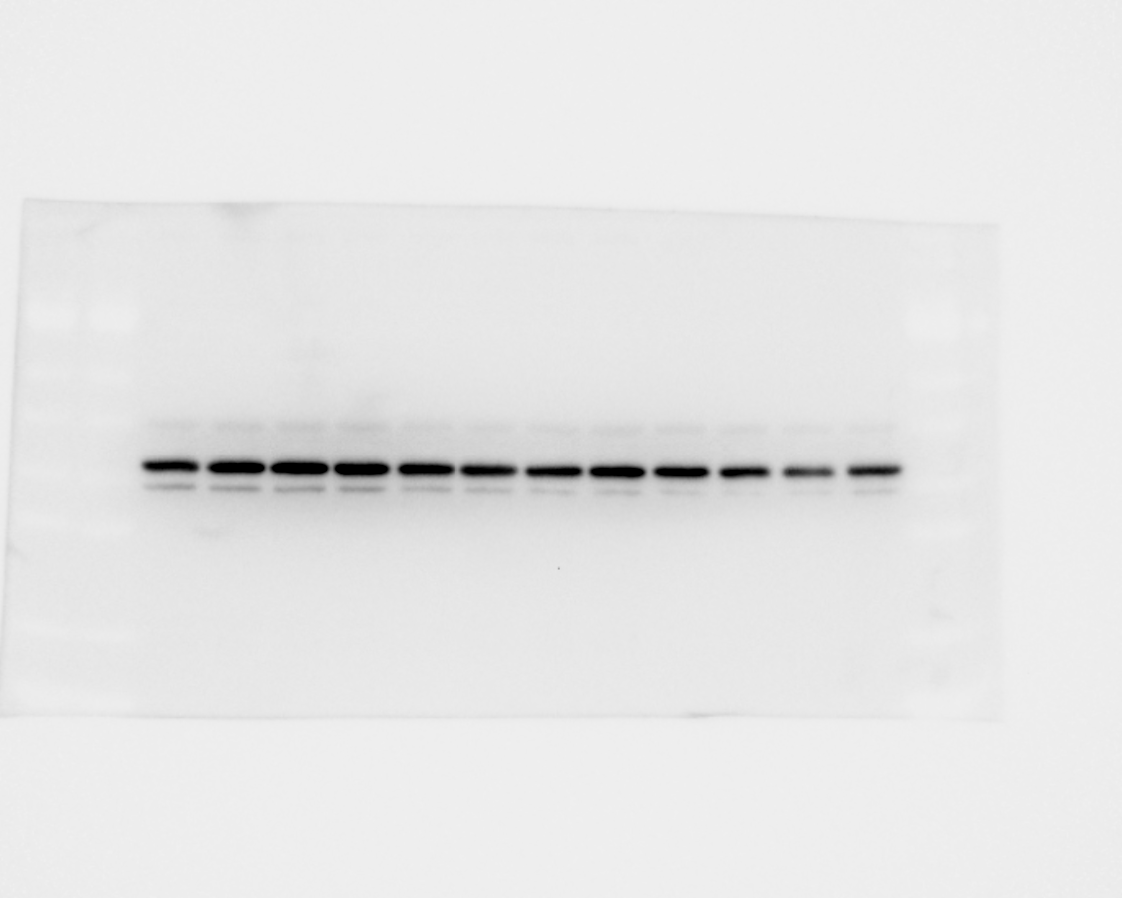

Supplement: Supplementary file 2 [file DataSheet1.ZIP › Syntenin/ljj 2024-07-31 15h30m22s(Chemiluminescence).tif]

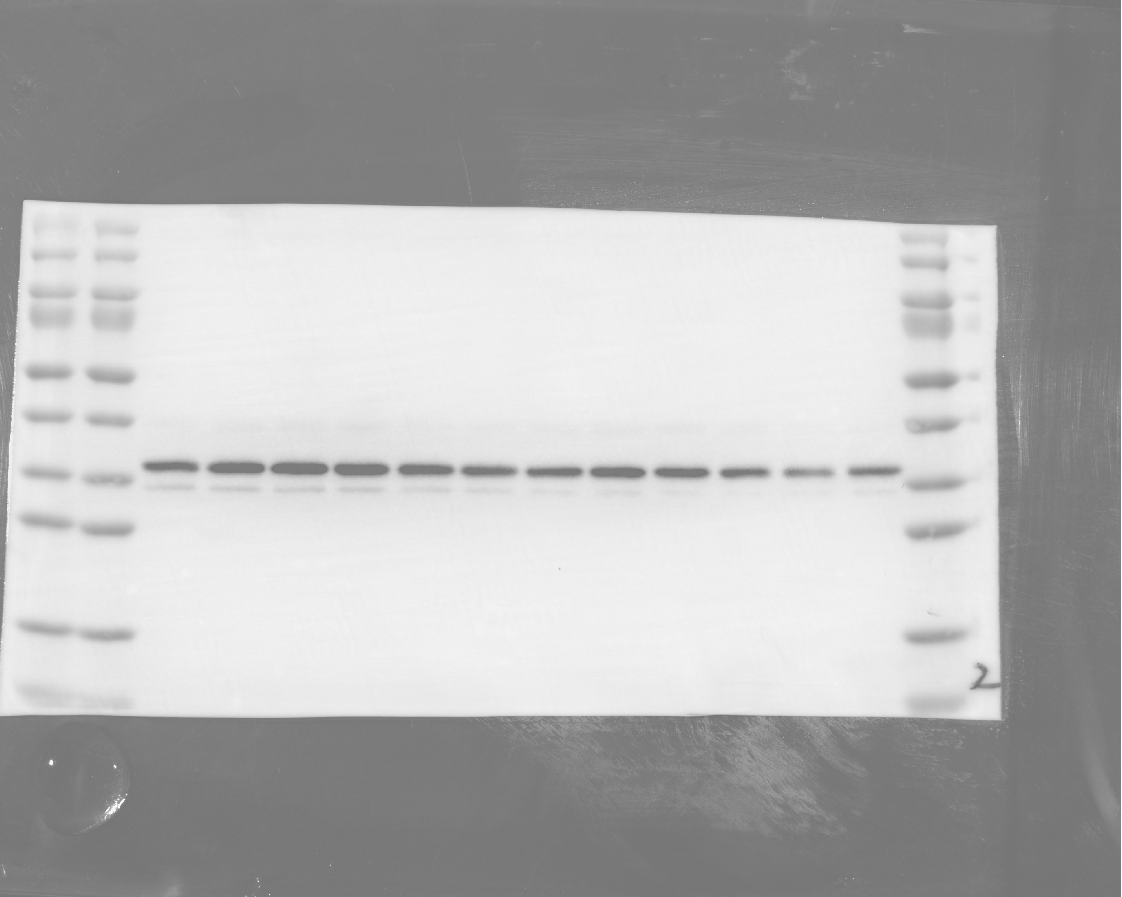

Supplement: Supplementary file 2 [file DataSheet1.ZIP › Syntenin/ljj 2024-07-31 15h31m12s(Composite).tif]

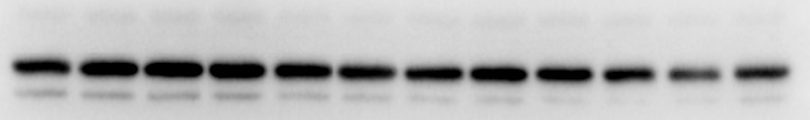

Supplement: Supplementary file 2 [file DataSheet1.ZIP › Syntenin/Syntenin.tif]

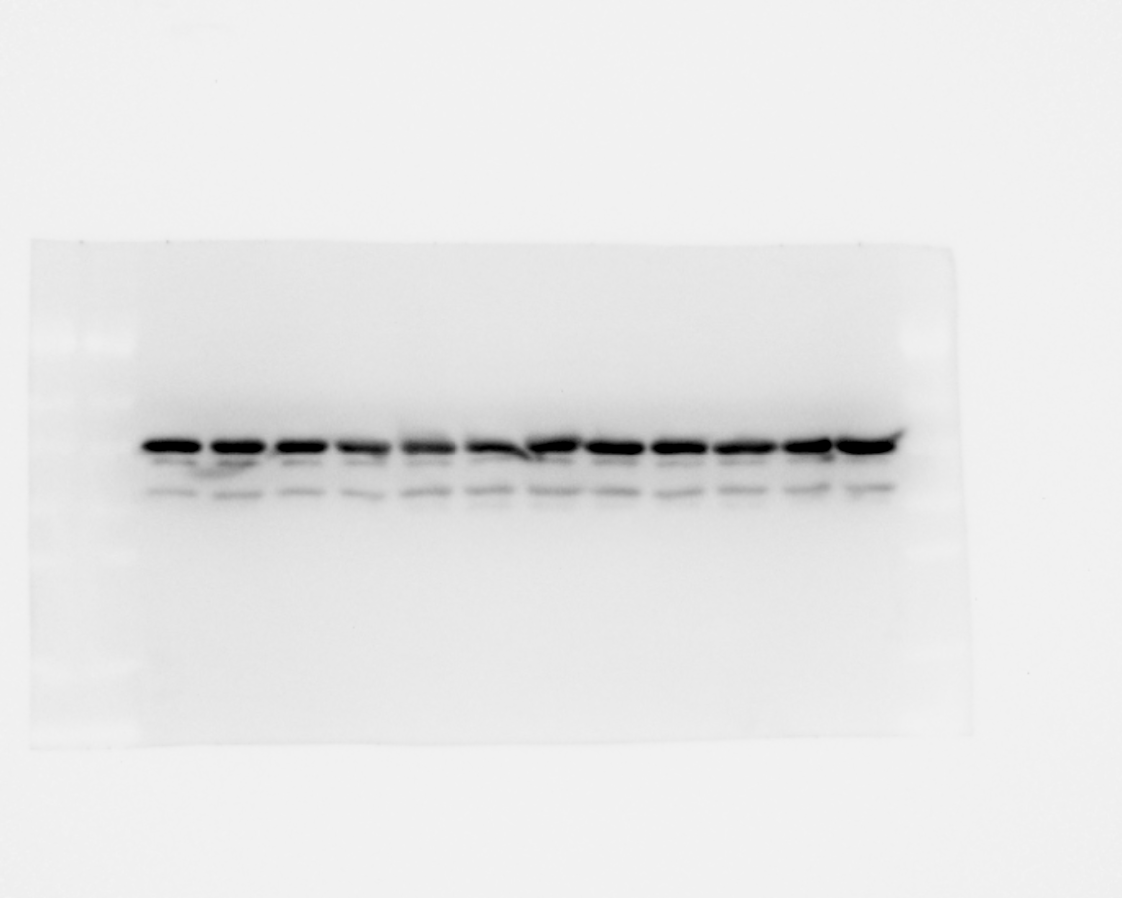

Supplement: Supplementary file 2 [file DataSheet1.ZIP › TSG101/ljj 2024-07-31 15h28m10s(Chemiluminescence).tif]

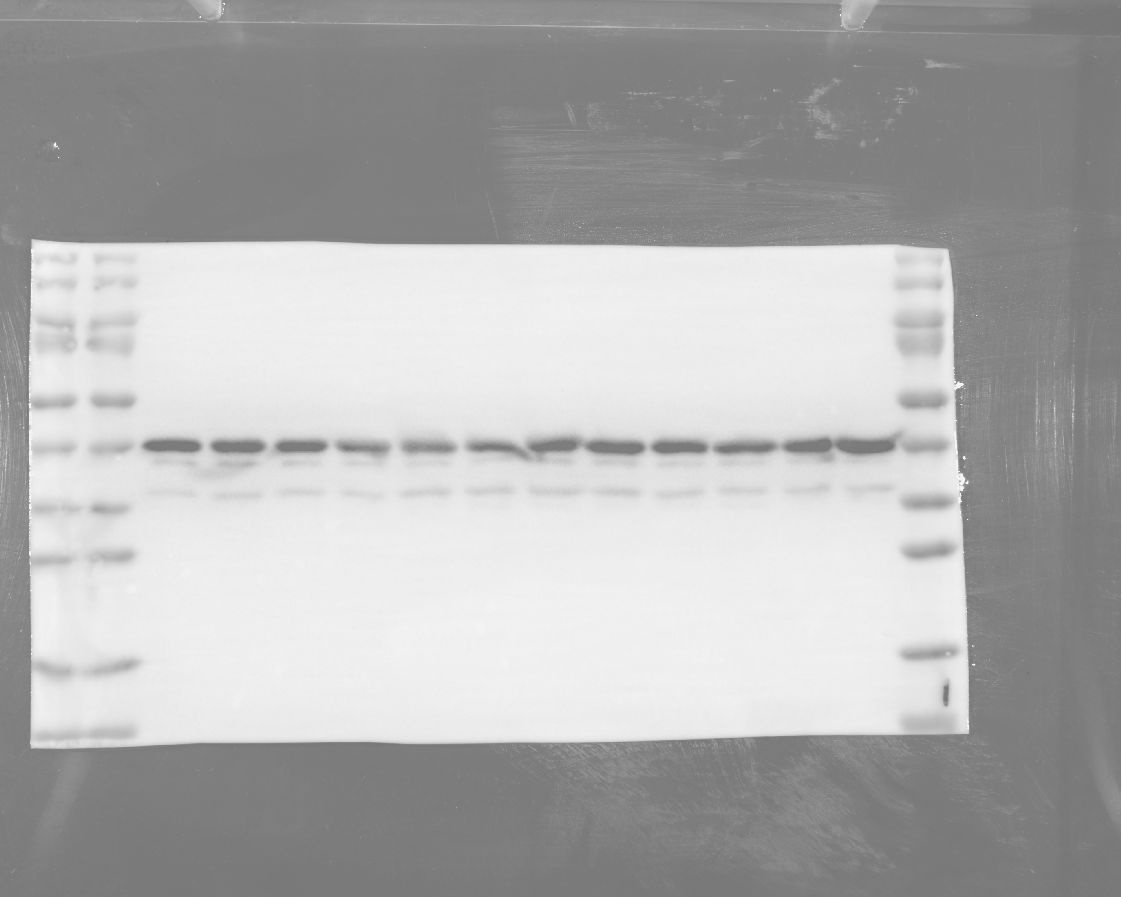

Supplement: Supplementary file 2 [file DataSheet1.ZIP › TSG101/ljj 2024-07-31 15h29m03s(Composite).tif]

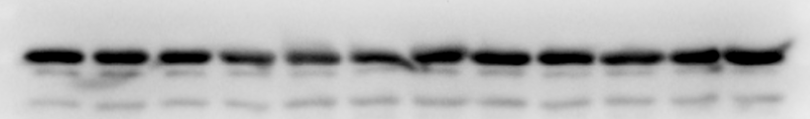

Supplement: Supplementary file 2 [file DataSheet1.ZIP › TSG101/TSG101.tif]

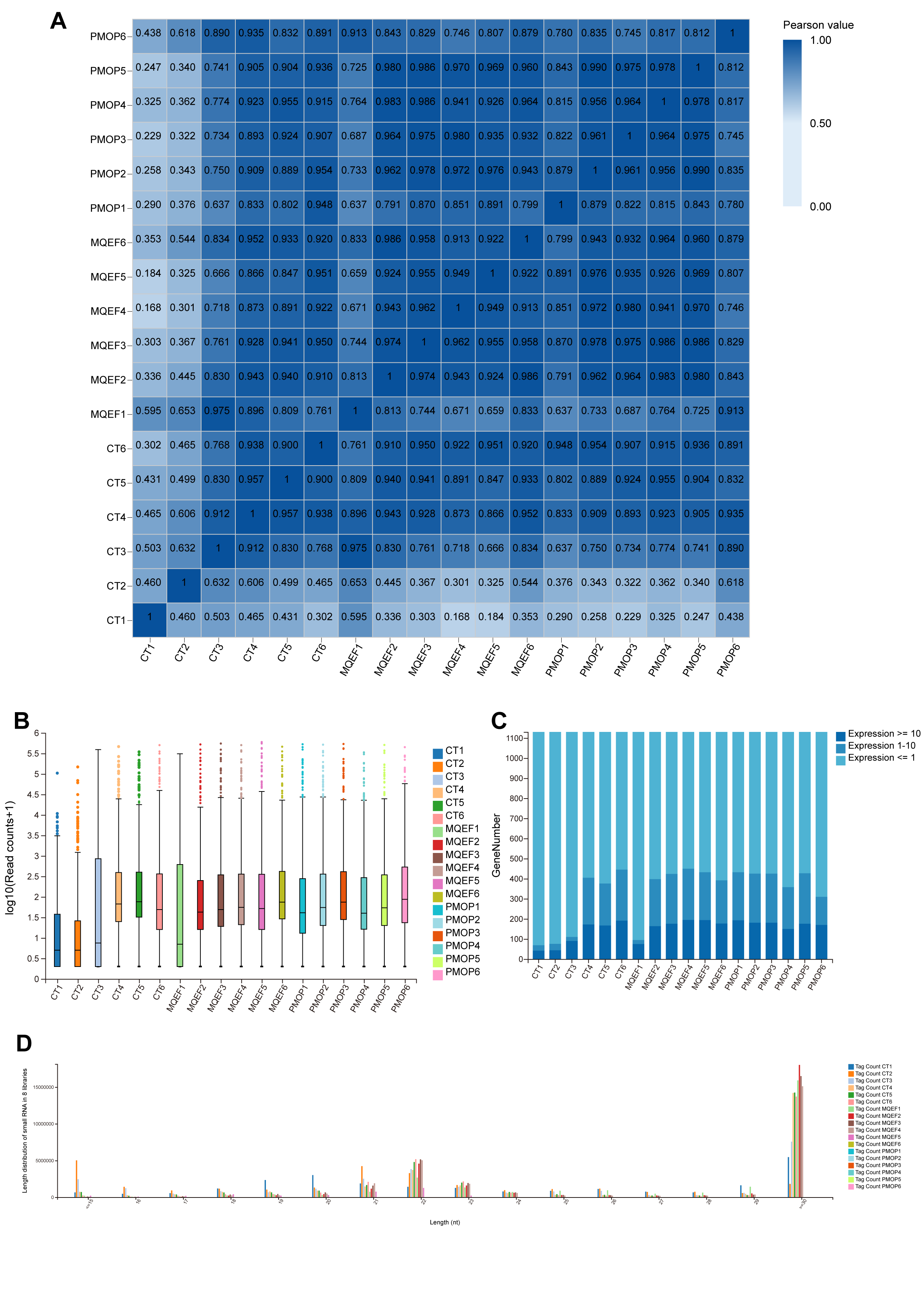

Supplement: Supplementary file 3 [file Image2.TIF]

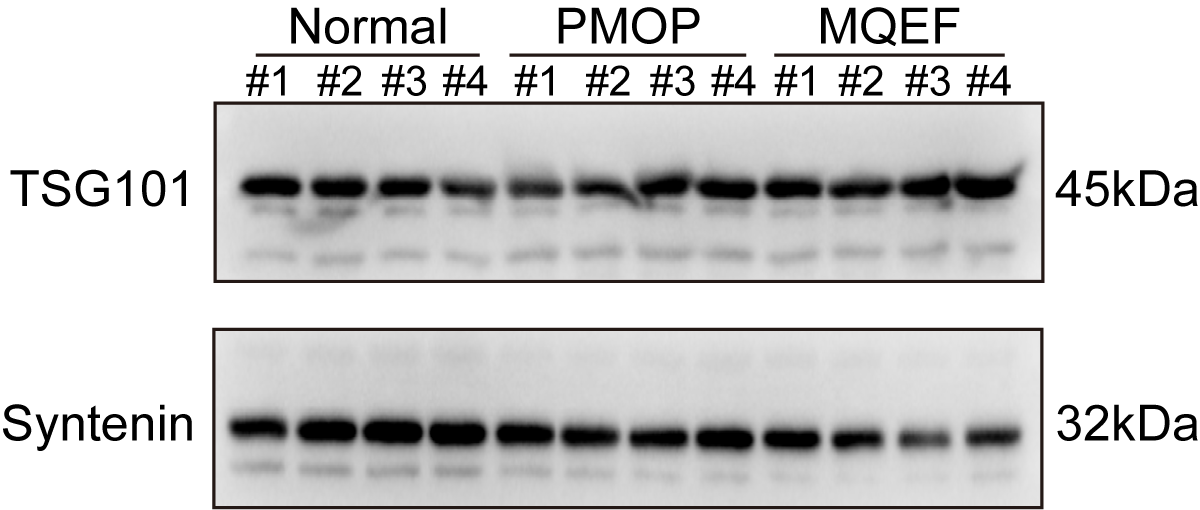

Supplement: Supplementary file 4 [file Image1.TIF]
